# Health

**Published:** 1873-04

**Authors:** 


### HOW TO EAT WISELY.

As a universal rule in health, and, with very rare exceptions, in disease, that is best to be eaten which the appetite craves or the taste relishes.

Persons rarely err in the quality of the food eaten; nature's instincts are the wise regulators in this respect.

The great sources of mischief from eating are three: Quantity, Frequency, Rapidity; and from these come the horrible dyspepsias which make of human life a burden, a torture, a living death.

Rapidity.—By eating fast, the stomach, like a bottle being filled through a funnel, is full and overflowing before we know it. But the most important reason is, the food is swallowed before time has been allowed to divide it in sufficiently small pieces with the teeth; for, like ice in a tumbler of water, the smaller the bits are, the sooner are they dissolved. It has been seen with the naked eye, that if solid food is cut up in pieces small as half a pea, it digests almost as soon, without being chewed at all, as if it had been well masticated. The best plan, therefore, is for all persons to thus comminute their food; for even if it is well chewed, the comminution is no injury, while it is of very great importance in case of hurry, forgetfulness, or bad teeth. Cheerful conversation prevents rapid eating.

Frequency —It requires about five hours for a common meal to be dissolved and pass out of the stomach, during which time this organ is incessantly at work, when it must have repose, as any other muscle or set of muscles, after such a length of effort. Hence persons should not eat within less than a five hours' interval. The heart itself is at rest more than one third of its time. The brain perishes without repose. Never force food on the stomach.

All are tired when night comes; every muscle of the body is weary and looks to the bed; but just as we lie down to rest every other part of the body, if we, by a hearty meal, give the stomach five hours' work, which, in its weak state, requires a much longer time to perform than at an earlier hour of the day, it is like imposing upon a servant a full day's labor just at the close of a hard day's work; hence the unwisdom of eating heartily late in the day or evening; and no wonder it has cost many a man his life. Always breakfast before work or exercise.

No laborers or active persons should eat an atom later than sun-down, and then it should not be over half the midday meal. Persons of sedentary habits or who are at all ailing, should take absolutely nothing for supper beyond a single piece of cold stale bread and butter, or a ship-biscuit, with a single cup of warm drink. Such a supper will always give better sleep and prepare for a heartier breakfast, with the advantage of having the exercise of the whole day to grind it up and extract its nutriment. Never eat without an inclination.

Quantity.—It is variety which tempts to excess; few will err as to quantity who will eat very slow. Take no more than a quarter of a pint of warm drink, with a piece of cold stale bread and butter, one kind of meat, and one vegetable, or one kind of fruit. This is the only safe rule of general application, and allows all to eat as much as they want.

Cold water at meals instantly arrests digestion, and so will much warm drink; hence a single tea-cup of drink, hot or cold, is sufficient for any meal.

For half an hour after eating sit erect, or walk in the open air. Avoid severe study or deep emotion, soon after eating. Do not sit down to a meal under great [illegible]rief or surprise, or mental excitement.

